# Investigating the Role of Glutamate and GABA in the Modulation of Transthalamic Activity: A Combined fMRI-fMRS Study

**DOI:** 10.3389/fphys.2017.00030

**Published:** 2017-01-31

**Authors:** Nathalie Just, Sarah Sonnay

**Affiliations:** ^1^CIBM-AIT core, Ecole Polytechnique Fédérale de LausanneLausanne, Switzerland; ^2^University Hospital MünsterMünster, Germany; ^3^LIFMET, Ecole Polytechnique Fédérale de LausanneLausanne, Switzerland

**Keywords:** fMRS, BOLD, resting state, barrel cortex, thalamus, glutamate, GABA

## Abstract

The Excitatory-Inhibitory balance (EIB) between glutamatergic and GABAergic neurons is known to regulate the function of thalamocortical neurocircuits. The thalamus is known as an important relay for glutamatergic and GABAergic signals ascending/descending to/from the somatosensory cortex in rodents. However, new investigations attribute a larger role to thalamic nuclei as modulators of information processing within the cortex. In this study, functional Magnetic Resonance Spectroscopy (fMRS) was used to measure glutamate (Glu) and GABA associations with BOLD responses during activation of the thalamus to barrel cortex (S1BF) pathway at 9.4T. In line with previous studies in humans, resting GABA and Glu correlated negatively and positively respectively with BOLD responses in S1BF. Moreover, a significant negative correlation (*R* = −0.68, *p* = 0.0024) between BOLD responses in the thalamus and the barrel cortex was found. Rats with low Glu levels and high resting GABA levels in S1BF demonstrated lower BOLD responses in S1BF and high amplitude BOLD responses in the thalamus themselves linked to the release of high GABA levels during stimulation. In addition, early analysis of resting state functional connectivity suggested EIB controlled thalamocortical neuronal synchrony. We propose that the presented approach may be useful for further characterization of diseases affecting thalamocortical neurotransmission.

**HIGHLIGHTS**

- Thalamic and barrel cortex fMRS, BOLD, and resting state fMRI were conducted.
- [GABA] inversely correlated with BOLD responses in S1BF.
- The stimulated thalamic neurochemical profile differed from that of S1BF.
- Regulation of functional connectivity between thalamus and cortex was suggested.

## Introduction

There is a growing body of evidence showing that both excitatory and inhibitory neurons govern the hemodynamic response to increased neuronal activity (Kocharyan et al., 2008; Enager et al., 2009; Logothetis et al., 2010; Lecrux et al., 2011) at the same time as they need “fuel” to work. As a consequence of this activity, neurons release glutamate (Glu), and γ-amminobutyric acid (GABA) neurotransmitters. Glu and GABA as well as other metabolites (Lactate, Aspartate, Glucose..) play a significant role in modulating brain activity during both stimulus-induced activity and “intrinsic” ongoing activity (Duncan et al., 2014). More than 10 years ago, Chen et al. (2005) demonstrated that GABA enhancement can decrease Blood Oxygen Level dependent (BOLD) signal amplitudes in the rat forepaw cortex. It is now well-admitted that resting state GABA concentrations correlate negatively with Blood Oxygen Level dependent responses in various regions of the human brain (Northoff et al., 2007; Donahue et al., 2010; Muthukumaraswamy et al., 2012; Bednařík et al., 2015). Morover, the modulation of neuronal activity by resting glutamate concentrations was also shown (Kapogiannis et al., 2013) although studies examining the relationship were sparser.

Another interesting approach for an improved understanding of neuroimaging signals may be to investigate how neuronal activity in a specific region of the brain influences the rest of its interconnected network. In particular, derivation of metabolic relationships between different regions in a neural network may be informative. Duncan et al. (2011) assessed functional connectivity between regions of the human anterior cingulate cortex (ACC) and related BOLD responses to [Glu] in one region but not the other thus demonstrating the influence of deactivation of one region on activation of the other through a glutamatergic pathway. Although the number of studies investigating how excitatory and inhibitory neurotransmitters mediate BOLD responses is growing, the potential of this relatively new approach remains to be explored.

In rodents, the thalamus is recognized as one of the most important brain areas for driving cortical processing (Devor et al., 2005; Poulet et al., 2012; Feldmeyer et al., 2013). Thalamic nuclei such as the ventral posteromedial thalamic nucleus (VPM) were often described as important relays for glutamatergic and GABAergic functional information processing of the ascending (and descending) response to whisker or forepaw stimulations. Notably, in models of absence epilepsy, the thalamus revealed to be more than a passive resonator for spike-wave-discharges maintenance while directional non-linear couplings between cortex and thalamus were suggested in conjunction with several glutamatergic and GABAergic receptor dysfunction (Lüttjohann and van Luijtelaar, 2015). Other pathologies such as schizophrenia could also originate from thalamic dysregulation and functional disconnectivity again linked to excitatory-inhibitory balance (EIB) dysfunction (Behrendt, 2006). In particular, several optogenetics studies suggested that glutamatergic and GABAergic modulations of neural activity could benefit the investigation of diseases affecting thalamo-cortical neurotransmission. In order to examine this proposition under pathological conditions, normal biochemical modulatory activities between cortex and thalamus must be characterized.

In the present study, the neurochemical profiles within the barrel cortex (S1BF) and the thalamus of rats before and during stimulation of the trigeminal nerve as well as BOLD responses were measured. A correlation study was then conducted to characterize associations between BOLD, [Lac], [Glu], and [GABA] in both thalamic and cortical structures and between them. We expected to verify that BOLD responses and [GABA] were negatively correlated within cortical and subcortical areas while positive correlations between BOLD and stimulation-induced changes in glutamate levels (Δ[Glu]) as well as lactate (Δ[Lac]) were envisaged in cortex. The influence of glutamate and GABA on thalamocortical neurotransmission were discussed.

## Materials and methods

### Animals

All studies were performed following the approval of Service de la consommation et des affaires vétérinaires du canton de Vaud (Switzerland) and according to the federal guidelines of the Animal Care and approved by the local authority (EXPANIM-SCAV). Male Sprague-Dawley rats (*n* = 15, 350 ± 40 g; Charles River, L'Arbresle, France) under isoflurane anesthesia (2–3%) vaporized in 30% O_2_ in air were intubated, and mechanically ventilated. Two femoral arteries and one femoral vein were catheterized for blood gas sampling and blood pressure measurements as well as α-chloralose (an initial intravenous dose of 80 mg/kg was administered followed by a continuous intravenous infusion of 27 mg/kg/h at a rate of 2 ml/h) and pancuronium administrations. Respiration rate was monitored through a pillow (SA Instruments, Stony Brook, NY, USA) placed underneath each rat. Temperature was measured using a rectal sensor and regulated via control of the temperature of water flowing through tubing covering the body of each rat and linked to a temperature-regulated bain-marie. Less than 300 μl of arterial blood were sampled every 30 min and blood parameters directly measured using an AVL blood gas analyzer (Dotmed, USA). Mean Arterial blood pressure (MABP) was measured continuously using a transducer attached to the femoral artery catheter. Body temperature and blood parameters were maintained at physiological levels (T = 37.5°C ± 0.5°C; pH = 7.4 ± 0.05, pCO2 = 39.7 ± 7 mmHg and MABP = 148.9 ± 11 mmHg) throughout each experiment. An intravenous femoral injection of Pancuronium Bromide (Sigma, Switzerland) of 0.7 ml per hour was performed to minimize tremors. Rats were positioned in a dedicated stereotactic holder equipped with ear and bite bars which was tilted in the magnet (30–45°) for a better positioning of voxels for fMRS over the barrel cortex. fMRS measurements were conducted sequentially in thalamus and S1BF followed by BOLD fMRI in 15 rats. The resting-state fMRI analysis is presented as supplementary data in the present study.

### Trigeminal nerve stimulation (TGN)

Electrodes were percutaneously inserted in the left infraorbital nerve. Electrical stimulation of the left trigeminal nerve (TGN) was performed using an external stimulator (WPI, Stevenage, UK) as described in Just et al. (2010). The paradigm of stimulation (1 minOFF–1 minON…) for both fMRI and fMRS was repeated for 32 min with pulse duration of 0.5 ms, stimulation frequency of 1Hz and stimulation current amplitude of 2 mA (Sonnay et al., 2015).

### Magnetic resonance experiments

**Figure 1:**
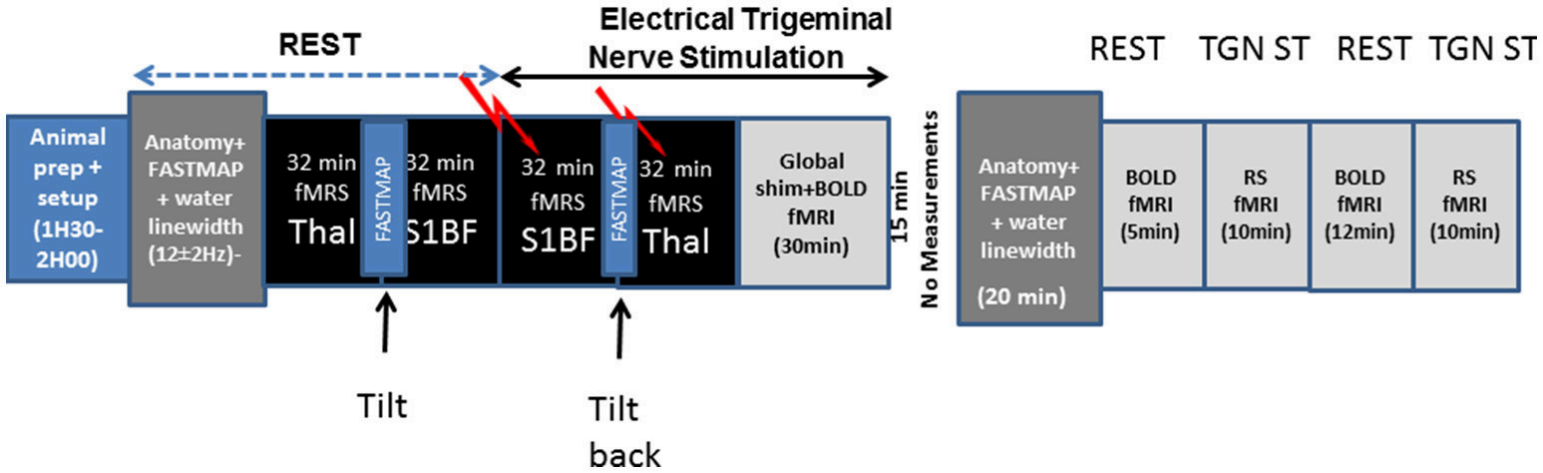
**Schematical description of the experimental setup used in the present study and timings**.

Experiments were described schematically in Figure 1. Experiments were performed on an actively shielded 9.4T/31 cm bore magnet (Agilent, USA) with 12 cm gradients and a surface coil. Shims were adjusted using FAST(EST)MAP (Gruetter and Tkác, 2000).

### Functional MR spectroscopy

Localized proton spectroscopy was performed using Spin Echo Full Intensity Acquired Localized Sequence (SPECIAL, TE = 2.8 ms, TR = 4 s) (Mlynárik et al., 2006) in 15 rats. The voxel of interest for the thalamus was chosen by reference to the Paxinos and Watson atlas (Paxinos and Watson, 1998) so that it encompasses the VPM and POm structures (*n* = 12). Its size was 3 × 3 × 4 mm^3^ and it was shimmed down to a linewidth of 12 ± 2Hz. In the barrel cortex, the VOI size was 1.5 × 3 × 5 mm^3^ and was shimmed down to a linewidth of 10 ± 2Hz (*n* = 15). For each VOI, ^1^H spectra were acquired during 32-min of rest followed by 32 min of TGN stimulation corresponding to 480 scans (30 × 16) per period. The water signal was suppressed using the VAPOR module containing a series of seven 25 ms asymmetric variable power RF pulses with optimized relaxation delays. To improve the signal localization, three modules of outer volume saturation (OVS) were interleaved with the water suppression pulses. The raw ^1^HMRS spectra corrected for frequency drift and summed were used for LCModel analysis with a basis set of 21 simulated metabolites (Macromolecules: Mac; Scyllo-inositol: Scyllo; Ala: Alanine; Ascorbate: Asc; Aspartate: Asp; β-hydroxybutyrate: bHb; Glycerophosphocholine: GPC, Phosphocholine: PCho; Creatine: Cr; Phosphocreatine; γ-aminobutyric acid: GABA; Glucose: Glc; Glutamine: Gln; Glutamate: Glu; Glutathione: GSH; myo-inositol: Ins; Lactate: Lac; N-acetylaspartate: NAA; *N*-Acetylaspartylglutamic acid:NAAG; Phosphatidylethanolamines: PE; Taurine: Tau). Absolute metabolite concentrations were obtained using unsuppressed water signal (8 averages) as an internal reference. The Cramer–Rao lower bounds were used as a reliability measure of the metabolite concentration estimates. Higher concentration metabolites (Glu, NAA, Ins, Tau, PCr, Cr) with Cramer-Rao lower bounds (CRLB) under 10% were considered to be reliably quantified whereas for Glc, GABA, Lac, Asp CRLB under 30% were considered acceptable and were kept for further analysis.

Neurochemical profiles in thalamus and S1BF of the same rat were acquired serially for 12 rats starting with the thalamic nuclei and S1BF at rest followed by successive 32-min TGN stimulation periods for S1BF and thalamus. A 15-min period of time was left before acquiring in S1BF at rest, which was used to tilt the holder and perform further adjustments (morphological T2 imaging and Shim) as well as to return the holder to initial position between acquisitions in S1BF and thalamus. A one-way ANOVA test with Bonferroni correction was used to compare metabolite concentrations at rest and during stimulation. The significance level was set at 0.05. All the results are presented as Mean ± SEM. A two-way ANOVA test with Bonferroni correction was performed to compare Glu, GABA, Lac, Glc, and Gln between thalamus and S1BF brain structures during ON (stimulation) and OFF (rest) measurement times.

### BOLD fMRI

BOLD responses in the barrel cortex and thalamus were measured post-fMRS measurements using single shot gradient echo EPI in 15 rats (7 extra rats underwent BOLD fMRI only, a total of 22 rats underwent BOLD–fMRI). **First** and second order shims were adjusted using FAST(EST)MAP (Gruetter and Tkác, 2000) resulting in water linewidths of 12 ± 3 Hz in a 216 μl (6 × 8 × 4.5 mm^3^) volume. After echo re-alignment using a reference scan, BOLD responses were assessed using single shot gradient echo EPI (TR/TE = 2500–2000/25 ms; FOV = 20 × 20 mm; matrix = 64 × 64; slice thickness = 1 mm; 8 slices, Bandwidth = 325 KHz, 900 volumes).

### Data analysis

Images were analyzed with SPM8 (Matlab; The Mathworks; Natick, USA; Statistical Parametric Mapping, www.fil.ion.ucl.ac.uk/spm/) using the general Linear model (GLM) analyzing each voxel independently and creating a parametric map of statistical significance (Friston et al., 1995). Gradient echo (GRE-EPI) time series were realigned, motion corrected, slice time corrected, normalized to each rat's anatomical images and spatially smoothed with a 3D Gaussian kernel (0.6 × 0.6 × 1 mm^3^). Within each analysis, the mean global intensities were mean scaled to an arbitrary value calculated within SPM8. The design model tested was a comparison between “off” and “on” conditions within each TGN stimulation paradigm. The paradigm was convolved with SPM's haemodynamic response function defined as a gamma-variate function and high pass-filtered. Residuals for the realigned rat movement were taken into account by submitting the realignment parameters (translations and rotations) as regressors. T-maps were calculated on a voxel by voxel basis. Thresholding criteria of 5 adjacent voxels, each with a T-score > 3.0 were used to identify regions of interest. Only clusters comprising at least 5 voxels were considered significant (*p* < 0.0001, corrected for multiple comparisons.

With STIMULATE (University of Minnesota, Minneapolis, USA) (Strupp, 1996), regions of interest (ROIs) over the activated primary somatosensory barrel field cortex (S1BF) were drawn with respect to the Paxinos and Watson's Atlas (Paxinos and Watson, 1998). ROIs were delineated from the thresholded t-map of each rat and had the same size as VOIs for 1H-MRS. A representative average time-course was recorded for each animal. When needed baseline correction was performed. T-maps were overlaid on single shot gradient echo EPI images.

### Correlations, robustness, and statistical analysis

Correlations were performed between BOLD responses in thalamus and S1BF, and [Glu] and [GABA] measured at rest and during stimulation in thalamus and S1BF. In addition BOLD responses were also correlated to changes in [Glu] and in [Lac] in S1BF only. Only metabolites with Cramer-Rao lower bounds (CRLB) under 30% were used for statistical analysis. To examine the association between BOLD responses and metabolites and between metabolites, the Shapiro-wilk test in SPSS 22 was performed for all the variables demonstrating *p*-values above 0.05 and therefore normality of each variable distribution.

The correlation analysis was performed within Origin (OriginLab version 9, Massachusetts, USA). Data were tabulated and scatter plots were obtained. Subsequently, a linear regression analysis was performed allowing to calculate the Pearson's r coefficient of correlation. Linear regression was peformed concomitantly with an ANOVA test with a threshold defined as 0.05. However, appropriate statistical analysis is required to perform multiple comparisons. With the conservative Bonferroni correction (Bretz et al., 2011) one would assume that all variables were independent which was not necessarily the case. Using a principal component analysis method within Origin, the number of effective comparisons (or eigenvalues) was determined as decribed by Cheverud et al. (1983). The adjusted threshold after Bonferroni correction was therefore *p* = α/4 with α = 0.05. Uncorrected *p*-values were denoted as *P* and were reported as correlating positively or negatively for *P* < 0.05. These values were comparable to values reported in the literature for a similar sample size (**Table 3**).

In order to examine the robustness of the correlation analysis performed in the present study, we further performed a Spearman- Rho correlation analysis within SPSS 22 since for small sample size data sets, a permulation analysis is automatically performed (Bretz et al., 2011). Finally, multiple linear regression analyses were performed in SPSS 22 for a sample size of 15 animals and for a sample size of 22 animals where the animal number was increased by adding 7 animals with CRLBs below 40% for Glu and GABA, since this type of regression analysis is valid for sample sizes of at least 20 subjects. Using a Kolmonorov-Smirnov test, the multivariate normality was checked again. To avoid over-fitting, a stepwise model analysis was used together with F statistics. The validity of the regression analysis was assessed and a Durbin-Watson test was included to check for autocorrelation and included a colinearity diagnostic. Homoscedasticity and normality of residuals were tested using standardized plots. To test the robustness of the multiple linear regression analysis Cook's and leverage tests were performed.

## Results

### Metabolic responses to prolonged TGN stimulations

**Figure 2:**
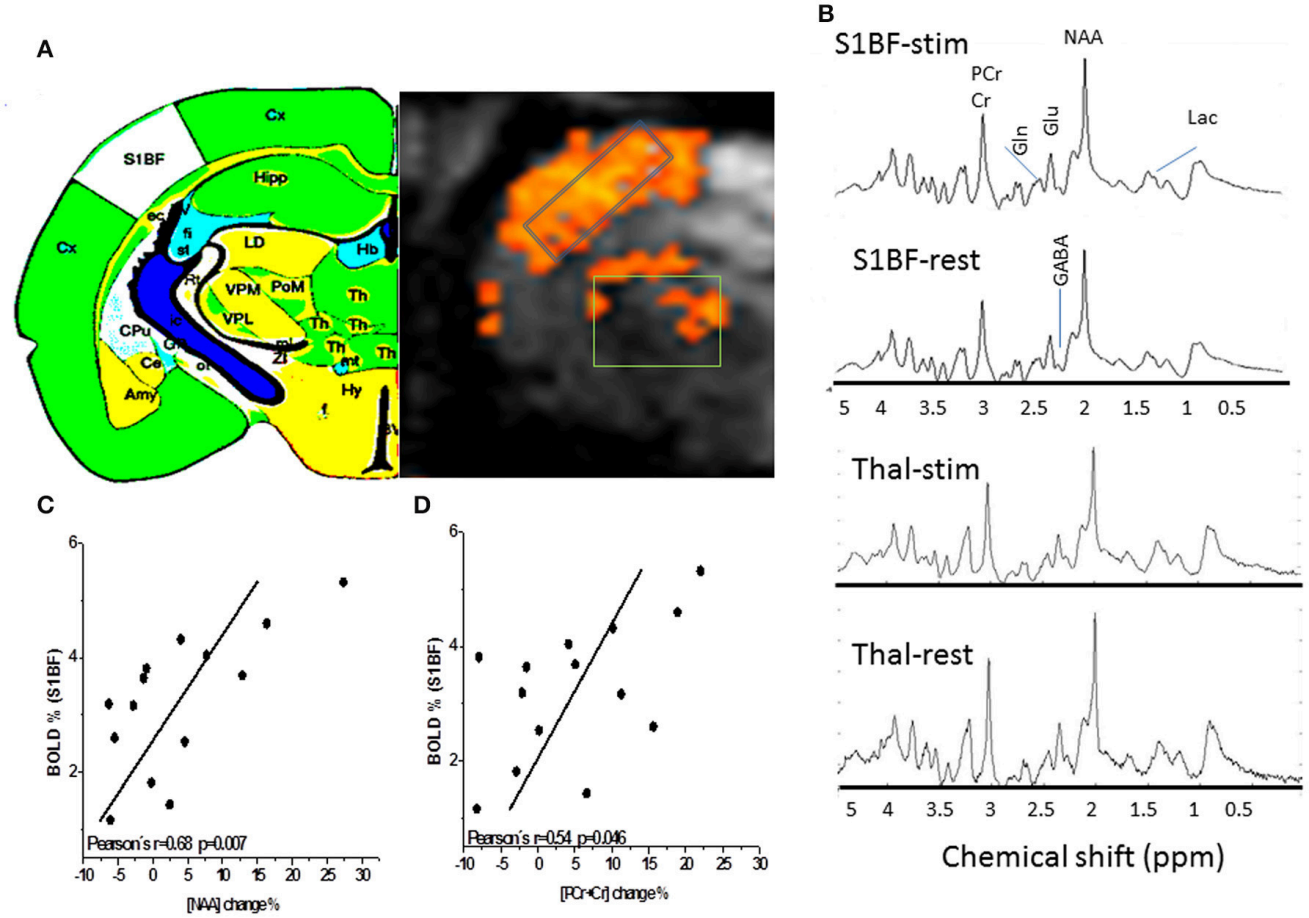
**Proton MR Spectroscopy of the rat barrel cortex and the thalamus. (A)** BOLD T map overlaid over gradient echo EPI and showing activations both in the barrel cortex and the thalamus. GABA, Glutamate, Glutamine, Glucose, and Lactate levels were measured at rest and during long stimulation periods both in the thalamus (*n* = 11) and in S1BF (*n* = 15) in the represented voxels of interest. The thalamic voxel of interest (36 μl) for fMRS in rats is depicted. In order to increase SNR levels for thalamic MRS acquisitions, the thalamic voxel encompassed several thalamic nuclei of interest [VPM, Reticular thalamic nucleus (RTN) and Posteromedian thalamic nucleus (POm)] that can be recognized by reference to the labeled map. Cx, Cortex; Th, Thalamus; S1BF, Primary somatosensory barrel field; Hb, Habenula; Hip, Hippocampus; Cpu, Caudate Putamen; Amy, Amygdala; **(B)** Examples of labeled spectra (mean spectra averaged over rat population and over time) in both structures acquired during stimulation and rest periods are shown. Increased and decreased levels of Glu and Lac levels during TGN stimulations can be visualized in each structure respectively. **(C)** and **(D)** BOLD effects result in a decrease of NAA and tCr (PCr+Cr) peak linewidths (increase in T2^*^). Changes in linewidths can also be reflected by a change in peak height reported here. Positive correlations (*r* = 0.68, *p* = 0.007 and *R* = 0.54, *P* = 0.046) were found between BOLD responses in S1BF and percent change of NAA and PCr+Cr peak height respectively. These changes may represent surrogate markers of BOLD responses but remain to be validated.

**Figure 3:**
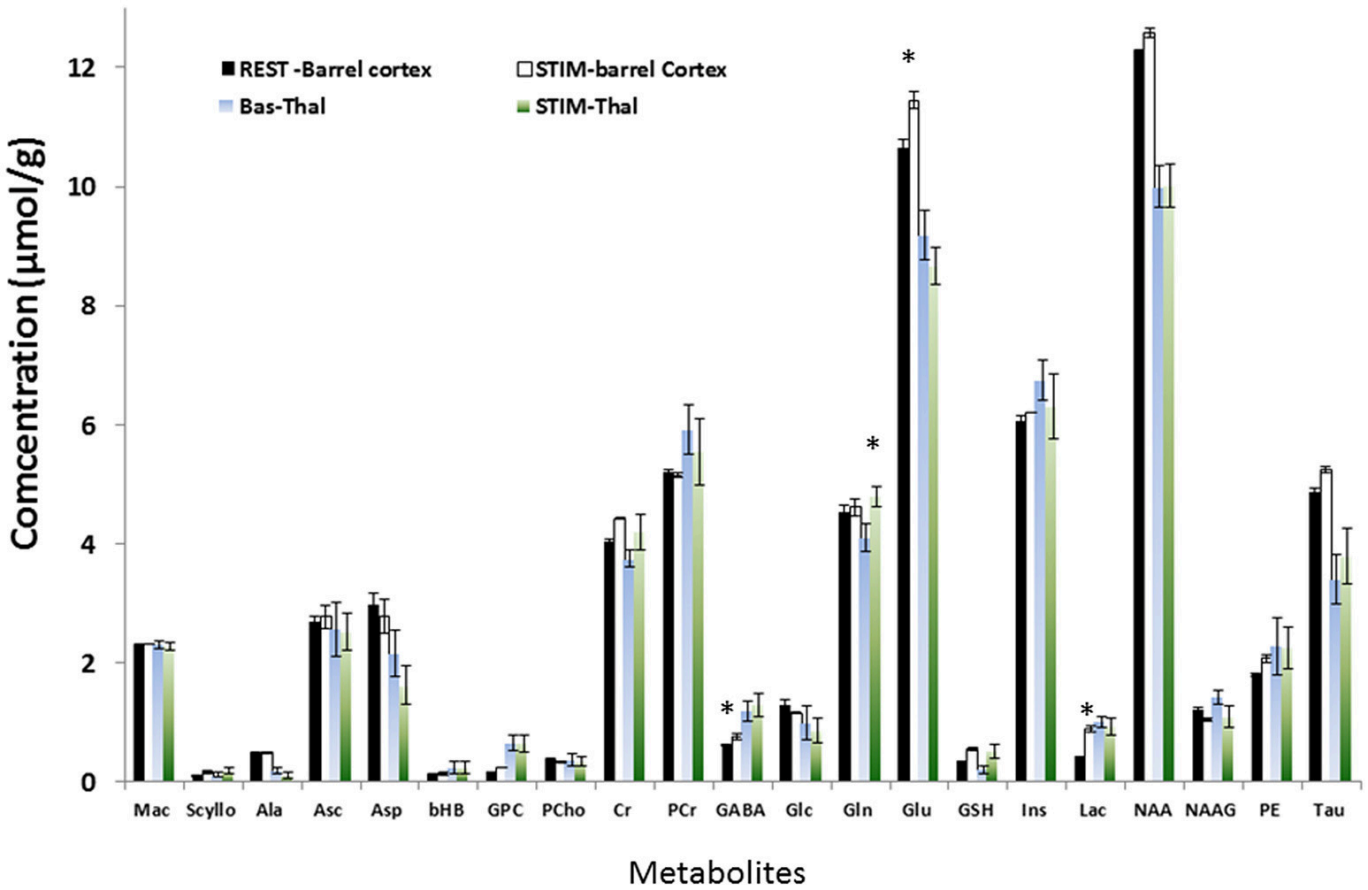
**Neurochemical profile of S1BF and thalamus during rest and TGN stimulation for 21 metabolites**. In S1BF, Lac, Glu, GABA levels were significantly higher during stimulation (*p* = 0.0015, *p* = 0.015, and *p* = 0.021 respectively, one-way ANOVA and Bonferroni correction). Glc and Asp demonstrated a tendency to decrease (*p* > 0.05). In Thalamus, Gln was significantly higher during TGN stimulation (*p* < 0.009, ANOVA +Bonferroni) while Glu only showed a strong tendency to decrease. Regional differences were demonstrated between thalamus and S1BF metabolites at rest and during stimulation using a two-way ANOVA test with Bonferroni correction. Significant differences between brain structures were found for Glu (*p* < 0.0001), Lac (*p* = 0.02), and GABA (*p* = 0.005). Changes in [Glu] and [GABA] in both S1BF and thalamus were significantly related to changes related to activation (*p* = 0.02).

The neurochemical profiles of the thalamus and the barrel cortex before and during TGN stimulation were obtained successively for each rat. High spectra SNR levels were reproducibly measured in S1BF (91 ± 8) and in the thalamus (62 ± 6).GABA, Glutamate, Glutamine, Glucose, and Lactate levels were measured at rest and during stimulation periods both in the thalamus (*n* = 12) and in S1BF (*n* = 15) in VOI represented in Figure 2A. In order to increase SNR levels for thalamic MRS acquisitions, the thalamic voxel encompassed several thalamic nuclei of interest [VPM, Reticular Thalamic nucleus (RTN), and Posteromedian Thalamic Nucleus (POm) that can be recognized by reference to the labeled map. Examples of labeled spectra (mean spectra averaged over rat population and over time)] in both structures acquired during stimulation and rest periods are shown (Figure 2B). Increased and decreased levels of Glu and Lac levels during TGN stimulations can be visualized in each structure respectively. Figures 2C,D illustrate positive correlations between BOLD changes and the percent change in amplitude of NAA and PCr+ Cr peaks respectively. As a consequence of BOLD effect, the linewidths of these peaks should decrease while the peak height should increase. No relationship was found in the thalamus. The neurochemical profiles (± standard error of the mean) are depicted (Figure 3) for each structure and each condition. In the present work, Glutamate and GABA concentrations were compared as well as Glucose and Glutamine in 15 rats (1 rat demonstrated lipid contamination during stimulation and was discarded from further analysis). In the barrel cortex, Lac, Glu, GABA levels were significantly higher during stimulation (*p* = 0.0015, *p* = 0.015, and *p* = 0.021 respectively, one-way ANOVA and Bonferroni correction). In the thalamus, Gln was significantly higher during TGN stimulation (*p* < 0.009, one-way ANOVA and Bonferroni correction). Although not significantly due to high variability across the rat population, glucose levels decreased in S1BF and thalamus. Regional differences were demonstrated between thalamus and S1BF metabolites at rest and during stimulation using a two-way ANOVA test with Bonferroni correction. Significant differences between brain structures were found for Glu (*p* < 0.0001), Lac (*p* = 0.02), and GABA (*p* = 0.005) during rest and stimulation periods. Changes in [Glu] and [GABA] in both S1BF and thalamus were significantly related to changes related to activation (*p* = 0.02).

### Relationships between bold responses, glutamate and GABA in S1BF, and thalamus

**Figure 4:**
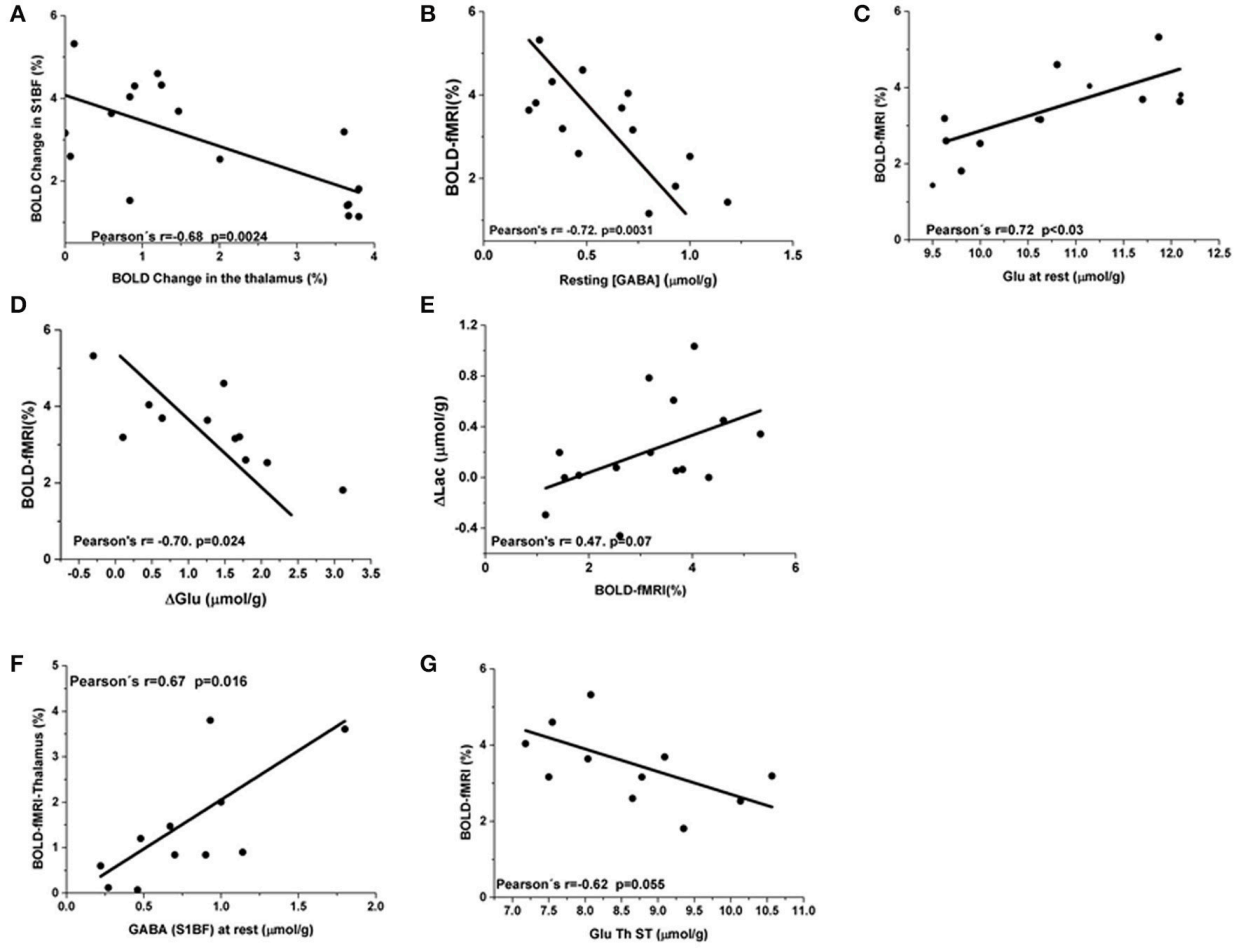
**BOLD responses correlations to [Glu] and [GABA] in S1BF. (A)** Negative correlation of Blood Oxygen Level dependent percent changes between the barrel cortex and the thalamus (Pearson's *r* = −0.68; *p* = 0.0024). **(B)** BOLD changes in the barrel cortex were negatively correlated to resting GABA levels (Pearson's coefficient = −0.72, *p* = 0.003). **(C)** A positive correlation between Glu levels at rest and BOLD responses was observed (Pearson's coefficient = 0.72, *P* < 0.03). **(D)** ΔGlu were negatively correlated to ΔBOLD (Pearson's coefficient = −0.70. *P* = 0.024). **(E)** A trend toward positive correlation between BOLD changes and Lac changes was also found (Pearson's *r* = 0.47; *P* = 0.07). Thalamic and S1BF BOLD responses correlations to [Glu] and [GABA]. In the thalamus, GABA levels were not correlated to thalamic BOLD responses neither at rest nor during activation. **(F)** At rest S1BF GABA levels demonstrated a positive interaction with thalamic BOLD approaching significance (Pearson's *r* = 0.67 and *P* = 0.02). **(G)** A negative correlation between S1BF BOLD and [Glu] during stimulation also approached significance (Pearson's *r* = 0.62 and *P* = 0.055).

Although rats underwent sequential prolonged MRS measurements in S1BF and thalamus successively during rest and stimulation periods, BOLD responses were still detected in S1BF and in the thalamus respectively. Unexpectedly, a negative correlation (Pearson's *r* = −0.68, *p* = 0.0024) was found as shown in Figure 4A, between BOLD responses in S1BF and thalamus. In order to investigate further this relationship, correlations between BOLD responses, [Glu] and [GABA] were performed within each structure and between structures. BOLD changes in the barrel cortex were negatively correlated to resting GABA levels (Pearson's coefficient = −0.72, *p* = 0.003) (Figure 4B). Resting Glu levels correlated positively with BOLD changes (Pearson's *r* = 0.72; *P* < 0.03, Figure 4C) and changes in glutamate (ΔGlu) were negatively correlated to BOLD (Pearson's coefficient = −0.70; *P* = 0.024) (Figure 4D). A trend toward positive correlation between BOLD changes and Lac changes (ΔLac) was also found but not significant (Pearson's *r* = 0.47; *P* < 0.08) (Figure 4E).

In the thalamus, resting or stimulated GABA levels were not correlated to thalamic BOLD responses. Resting S1BF GABA levels demonstrated a positive interaction with thalamic BOLD (Pearson's *r* = 0.67 and *P* = 0.016, Figure 4F). Resting thalamic Glu levels were not correlated with thalamic BOLD changes (Pearson's *r* = 0.57; *P* < 0.09) and with BOLD changes in the barrel cortex (Pearson's *r* = −0.55, *P* = 0.09). A negative correlation between S1BF BOLD and [Glu] during stimulation was also suggested (Pearson's *r* = 0.62 and *P* = 0.055, Figure 4G).

**Figure 5:**
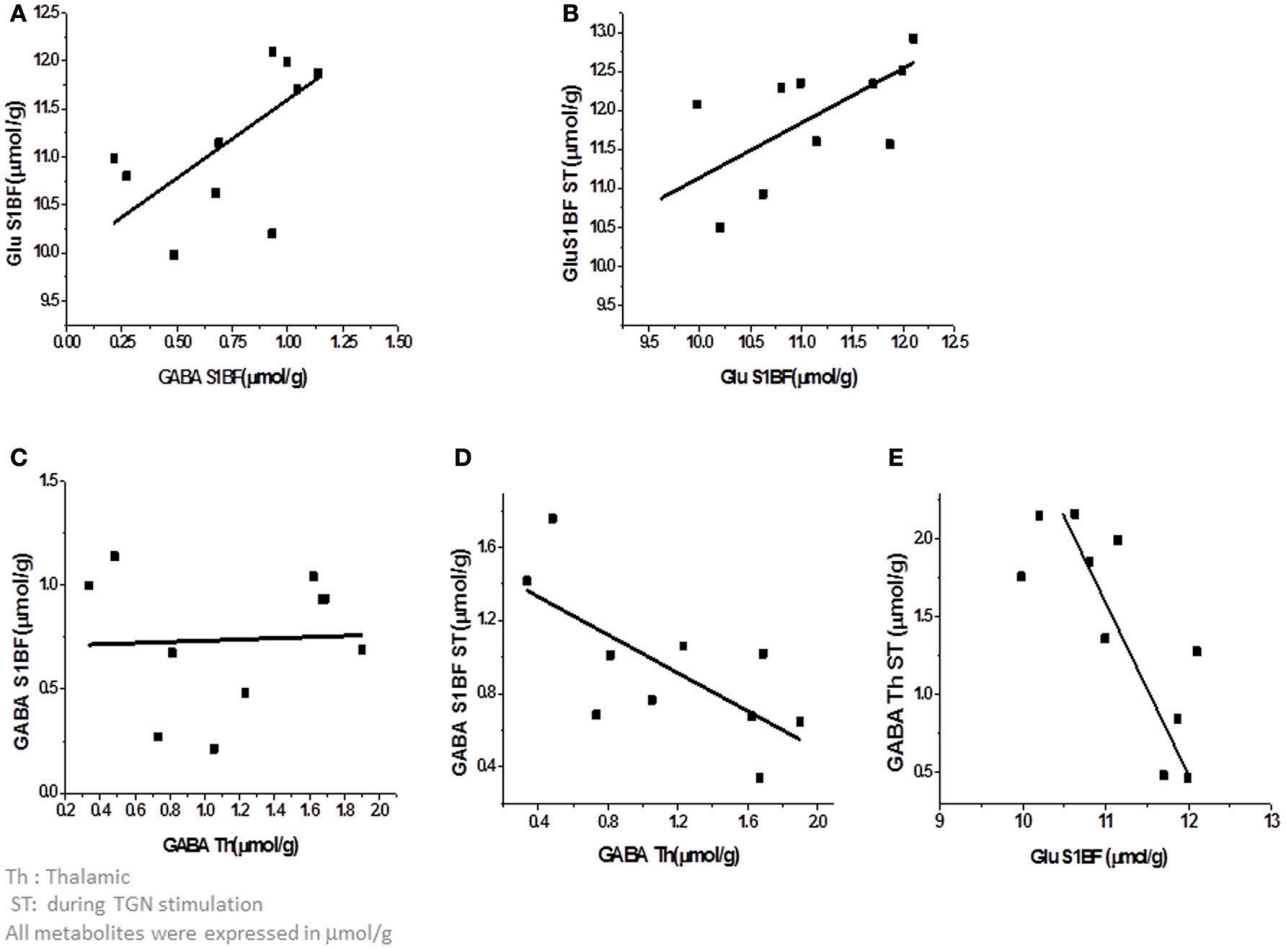
**Correlations between metabolites within and between the thalamus and the cortex. (A)** At rest, GABA and Glu levels in the barrel cortex were positively correlated (Pearson's *r* = 0.74; *P* = 0.014). **(B)** In S1BF, Glu levels at rest and during stimulation were correlated (Pearson's *r* = 0.67; *P* < 0.05) **(C,D)**. Thalamic GABA levels at rest were negatively correlated to barrel cortex GABA levels during stimulation (Pearson's *r* = −0.69; *P* = 0.024) but not at rest (Figure 4C). **(E)** Glu levels in S1BF at rest were strongly influenced by stimulated thalamic GABA levels.

Since the metabolic status of a structure may be predictive of its functional status including connectivity (Kapogiannis et al., 2013; Castro-Alamancos and Gulati, 2014), the relationships between [Glu] and [GABA] within and between structures were also investigated. As the energetic status of the whole brain is tightly controlled at rest, changes due to neuronal activity also involve changes between related structures. As reported in Table 1, during TGN stimulation, there was no correlation between [GABA] and [Glu] whereas at rest, GABA and Glu levels in the barrel cortex were positively correlated (Pearson's *r* = 0.74; *P* = 0.014, Figure 5A) and resting and stimulated [Glu] in S1BF were positively correlated (Figure 5B). On the other hand, resting thalamic GABA levels were negatively correlated to barrel cortex GABA levels measured during stimulation (Pearson's *r* = −0.69; *P* = 0.024) (Figure 5D) but not at rest (Figure 5C). However, Glu levels in S1BF at rest strongly influenced stimulated thalamic GABA levels (Figure 5D; Table 1). In S1BF, resting and activated Glu levels were correlated (Pearson's *r* = 0.67; *P* < 0.05, Figure 5E).

**Table 1:** **Glutamate and GABA concentrations correlations in S1BF and Thalamus at rest and during TGN Stimulations**.

		**R**	**E**	**S**	**T**
		**Glutamate thalamus**	**Glutamate S1BF**	**GABA Thalamus**	**GABA S1BF**
R	Glutamate thalamus	1	*r* = −0.06; *P* = 0.9	*r* = 0.40; *P* = 0.28	*r* = 0.55; *P* = 0.12
E	Glutamate S1BF	*r* = −0.06; *P* = 0.9	1	*r* = 0.05; *P* = 0.9	*r* = 0.74; *P* = 0.014
S	GABA Thalamus	*r* = 0.40; *P* = 0.28	*r* = 0.05; *P* = 0.9	1	*r* = 0.05; *P* = 0.89
T	GABA S1BF	*r* = 0.55; *P* = 0.12	*r* = 0.74; *P* = 0.014	*r* = 0.05; *P* = 0.9	1
S	Glutamate thalamus	*r* = 0.63; *P* = 0.07	*r* = −0.53; *P* = 0.11	*r* = 0.25; *P* = 0.52	*r* = −0.06; *P* = 0.9
T	Glutamate S1BF	*r* = −0.33; *P* = 0.34	*r* = 0.67; *P* = 0.049	*r* = 0.05; *P* = 0.9	*r* = 0.63; *P* = 0.07
I	GABA Thalamus	*r* = 0.41; *P* = 0.27	*r* = −0.78; *P* = 0.022	*r* = 0.40; *P* = 0.28	*r* = −0.47; *P* = 0.19
M	GABA S1BF	*r* = −0.029; *P* = 0.94	*r* = 0.3; *P* = 0.44	*r* = −0.7; *P* = 0.024	*r* = 0.28; *P* = 0.28
		**S**	**T**	**I**	**M**
		**Glutamate thalamus**	**Glutamate S1BF**	**GABA Thalamus**	**GABA S1BF**
S	Glutamate thalamus	1	*r* = −0.46; *P* = 0.21	*r* = 0.25; *P* = 0.52	*r* = 0.05; *P* = 0.90
T	Glutamate S1BF	*r* = −0.46; *P* = 0.21	1	*r* = −0.60; *P* = 0.115	*r* = −0.09; *P* = 0.81
I	GABA Thalamus	*r* = 0.25; *P* = 0.52	*r* = −0.60; *P* = 0.115	1	*r* = −0.51; *P* = 0.16
M	GABA S1BF	*r* = 0.05; *P* = 0.90	*r* = −0.09; *P* = 0.81	*r* = −0.51; *P* = 0.158	1

### Multiple linear regression analysis and robustness of the analysis

Table 2 presents the results of the Spearman-Rho correlation analysis. Results were in agreement with previous Pearson's correlation (*p*-values under 0.01 and 0.05).

Table 2Spearman–Rho correlation and statistics (^**^p < 0.01; ^*^p < 0.05).

**Table:** 

			**BOLD BC**	**Glu rest BC**	**Glu stim BC**	**Δglu BC BC**	**GABA rest BC**	**BOLD TH**	**GABA ST BC**
Spearman-Rho	BOLD BC	Correlations coefficient	**1.000**	0.701^**^	−0.198	−0.824^**^	−0.586*	−0.719^**^	0.150
		Sig. (2-sided)		**0.005**	0.497	**0.000**	**0.014**	**0.004**	0.610
		*N*	**15**	**15**	**15**	**15**	**15**	**15**	**15**
	Glutamate rest BC	Correlations coefficient	0.701^**^	**1.000**	0.420	−0.459	0.705^**^	−0.481	−0.099
		Sig. (2-sided)	**0.005**		0.135	0.098	**0.005**	0.081	0.736
		*N*	**15**	**15**	**15**	**15**	**15**	**15**	**15**
	Glutamate stimulation BC	Correlations coefficient	−0.198	0.420	**1.000**	0.473	0.033	0.235	−0.275
		Sig. (2-sided)	0.497	0.135		0.088	0.911	0.418	0.341
		*N*	**15**	**15**	**15**	**15**	**15**	**15**	**15**
	ΔGlu BC	Correlations coefficient	−0.824^**^	−0.459	0.473	**1.000**	−**0.556**	0.767^**^	0.092
		Sig. (2-sided)	**0.000**	0.098	0.088		**0.039**	**0.001**	0.753
		*N*	**15**	**15**	**15**	**15**	**15**	**15**	**15**
	GABA rest BC	Correlations coefficient	−0.586^*^	0.705^**^	0.033	−0.556^*^	**1.000**	−0.433	0.070
		Sig. (2-sided)	**0.014**	**0.005**	0.911	**0.039**		0.122	0.811
		*N*	**15**	**15**	**15**	**15**	**15**	**15**	**15**
	BOLD TH	Correlations coefficient	−0.719^**^	−0.481	0.235	0.767^**^	−0.433	**1.000**	0.145
		Sig. (2-sided)	0.004	0.081	0.418	0.001	0.122		0.620
		*N*	**15**	**15**	**15**	**15**	**15**	**15**	**15**
	GABA stimulation BC	Correlations coefficient	0.150	−0.099	−0.275	0.092	0.070	0.145	**1.000**
		Sig. (2-sided)	0.610	0.736	0.341	0.753	0.811	0.620	
		*N*	**15**	**15**	**15**	**15**	**15**	**15**	**15**

**Table:** 

			**GABA Th**	**GABA S1BF**	**GABA Th ST**	**GABA S1BF ST**	**Glu Th**	**Glu S1BF**	**Glu Th ST**	**GluS1BF ST**
Spearman-Rho	GABA Th	Correlations coefficient	**1.000**	0.080	0.449^*^	−0.412	0.309	0.069	0.216	0.111
		Sig. (2-sided)		0.731	**0.047**	0.064	0.185	0.766	0.347	0.632
		*N*	**15**	**15**	**15**	**15**	**15**	**15**	**15**	**15**
	GABA S1BF	Correlations coefficient	0.080	**1.000**	−0.379	0.373	0.521^*^	0.764^**^	0.093	0.163
		Sig. (2-sided)	0.731		0.099	0.096	**0.018**	**0.000**	0.688	0.479
		*N*	**15**	**15**	**15**	**15**	**15**	**15**	**15**	**15**
	GABA Th ST	Correlations coefficient	0.449^*^	−0.379	**1.000**	−0.304	0.067	−0.408	0.221	−0.455^*^
		Sig. (2-sided)	**0.047**	0.099		0.193	0.785	0.074	0.348	**0.044**
		*N*	**15**	**15**	**15**	**15**	**15**	**15**	**15**	**15**
	GABA S1BF ST	Correlations coefficient	−0.412	0.373	−0.304	**1.000**	0.236	0.438^*^	0.455^*^	0.367
		Sig. (2-sided)	0.064	0.096	0.193		0.316	**0.047**	**0.038**	0.102
		*N*	**15**	**15**	**15**	**15**	**15**	**15**	**15**	**15**
	Glu Th	Correlations coefficient	0.309	0.521^*^	0.067	0.236	**1.000**	0.388	0.806^**^	0.030
		Sig. (2-sided)	0.185	**0.018**	0.785	0.316		0.091	**0.000**	0.899
		*N*	**15**	**15**	**15**	**15**	**15**	**15**	**15**	**15**
	Glu S1BF	Correlations coefficient	0.069	0.764^**^	−0.408	0.438^*^	0.388	**1.000**	0.106	0.518^*^
		Sig. (2-sided)	0.766	**0.000**	0.074	**0.047**	0.091		0.646	**0.016**
		*N*	**15**	**15**	**15**	**15**	**15**	**15**	**15**	**15**
	Glu Th ST	Correlations coefficient	0.216	0.093	0.221	0.455^*^	0.806^**^	0.106	**1.000**	0.114
		Sig. (2-sided)	0.347	0.688	0.348	**0.038**	**0.000**	0.646		0.624
		*N*	**15**	**15**	**15**	**15**	**15**	**15**	**15**	**15**
	GluS1BF ST	Correlations coefficient	0.111	0.163	−0.455^*^	0.367	0.030	0.518^*^	0.114	**1.000**
		Sig. (2-sided)	0.632	0.479	**0.044**	0.102	0.899	**0.016**	0.624	
		*N*	**15**	**15**	**15**	**15**	**15**	**15**	**15**	**15**

*The Spearman-Rho coefficient of correlation between BOLD BC and ΔLac was 0.461 with p = 0.084 (n = 15)*.

Stepwise non-parameteric multiple linear regression analyis in a sample size of 15 animals revealed that resting Glutamate and GABA levels added significant explanatory power to the regression model. In addition, resting GABA and glutamate levels in the barrel cortex (BC) explained between 52.3 and 89% of the variance. The Durbin-watson test (D = 1.631) allowed to assume that there was no significant first order linear autocorrelation in the multiplelinear regression data. The *F*-Test is the test of significance of the multiple linear regression. The *F*-test of was highly significant (*F* = 19.063, *p* = 0.012 for Glu_rest_ and *p* = 0.001 for GABA_rest_,) thus it can be assumed that there is a linear relationship between the variables in our model. Since we have multiple independent variables in the analysis the Beta weights compared the relative importance of each independent variable in standardized terms. We found that a regression model encompassing BOLD and resting Glutamate and GABA levels had the highest impact on the regression (β = 0.955 and 0.383). Multicollinearity in our multiple linear regression model was higher than MC = 0.811 showing that there was no suspiscion of multicollinearity. Q-Q plots indicated that in our multiple linear regression analysis there was no tendency in the error terms. Finally, Cook's and centered leverage mean values were 0.223 ± 0.4 and 0.273 ± 0.2 showing that exclusion of data points wouldn't have changed the regression statistics substantially while the centered leverage value demonstrated that there was no significant influence of specific datapoints. The same analysis was performed with a sample size of 22 animals showing that resting glutamate levels only added significant explanatory power to the regression model explaining 80% of the variance confirmed by highest Beta weights (β = 0.896). Again, there were no significant influence of autocorrelations and multicollinearity (D = 2.3, MC = 0.655). F-tests were again highly significant (*F* = 44.837, *p* < 0.0001). Finally due to the slight increase in sample size, Cook#s and centered leverage values were further decreased 0.077 ± 0.258 and 0.048 ± 0.045.

## Discussion

In the present work, using fMRS and BOLD fMRI measurements in the thalamus and barrel cortex of rats, the modulation of neuronal activity- represented by BOLD responses- within and between these connected structures by GABA and Glu neurotransmitters was addressed with correlations. To the best of our knowledge, the present study is the first attempt to measure and compare functional hemodynamic and metabolic responses upon sensory stimulation of related brain structures in rats using MR-based methods. Although as discussed below, a number of technical challenges remain to be solved to validate the methodology proposed, the approach is potentially powerful for multi-regional investigations during brain activity and a better understanding of the underlying relationships between neurotransmitters and functional neuroimaging signals as well as functional connectivity.

### Confirmation of cortical BOLD-neurotransmitter relations in the activated region

Our study confirmed the cortical BOLD responses dependence on resting GABA levels as well as changes in Glu and Lac in response to stimulation but revealed that the direction of the correlations was specific to the system under analysis. In agreement with previous results obtained in the visual human cortex (Northoff et al., 2007; Donahue et al., 2010; Muthukumaraswamy et al., 2012; Bednařík et al., 2015), positive S1BF BOLD signals were negatively correlated to baseline S1BF GABA levels. In the human visual cortex, concomitant positive, and negative correlations with Cerebral Blood Flow (CBF) and vascular space occupancy (VASO) signals respectively, were interpreted as a greater influx of blood to the visual cortex region in participants with higher GABA levels in order to fuel the greater amount of excitatory activity required for overcoming the higher resulting inhibition. In S1BF, low [Glu]_rest_ predicted lower BOLD responses whereas lower Δ[Glu] predicted higher BOLD percent changes during TGN stimulation. Furthermore, Δ[Lac] suggested a positive correlation with BOLD fMRI responses (Pearson's *r* = 0.47 *P* = 0.07). These findings were in line with the study by Bednařík et al. (2015) where higher Glu changes (Δ[Glu]) as well as higher Lac changes (Δ[Lac]) predicted higher BOLD percent changes in the human visual cortex, interpreted as a response to increased energy demands of neuronal activation. Although, this study confirmed the existence of relationships between BOLD responses and both GABA at rest and Glu changes, the negative relationship with the latter was unexpected and difficult to interpret: If higher ΔGlu levels not compensating for the inherently higher GABA levels in S1BF of some rats and therefore leading to lower BOLD responses could represent a reasonable interpretation, null ΔGlu (at intercept) resulting in highest BOLD responses appeared contradictory to previous results. To date, the majority of fMRS studies reported weak mean Δ[Glu] ranging between 2 and 4% due to cortical stimulation (Mangia et al., 2007; Lin et al., 2012; Schaller et al., 2014). In these studies, fMRS was conducted after carefully positioning the VOI over the core activated area in BOLD maps for which fMRI acquisitions were optimized. In the present study, BOLD responses were measured after fMRS acquisitions. The position of the VOI in S1BF relied on previous work (Just et al., 2013) possibly missing the S1BF activated core and thus shifting the amplitudes of vascular and metabolic responses. However, positive changes in amplitudes of NAA and tCr peaks as a consequence of BOLD changes were detected as in Just et al. (2013) suggesting that the choice of VOI was adequate. Moreover, averaging the voxels yielding the highest statistically significant S1BF BOLD responses also revealed a negative correlation (not shown) with Δ[Glu] thus discarding effects related to thresholding. Weak changes in [Gln] in S1BF also corresponded to rats with highest BOLD responses and could not serve as an explanation. Bednařík et al. (2015) also found a non-zero Δ[Glu] intercept for BOLD = 0%. Although they point at different sensitivities of fMRI and fMRS signals and agree that larger populations need to be examined, their study and the present one point at other effects. In particular, the effects of other neuromodulators such as norepinephrine, noradrenaline, dopamine etc…also need to be taken into account in future studies and may explain these discrepancies (Fontanez and Porter, 2006; Qiao et al., 2007; Castro-Alamancos and Gulati, 2014). Moreover, the literature reported either positive (Enzi et al., 2012) or negative correlations between BOLD and baseline Glu concentrations, which depended on the health status of patients (Falkenberg et al., 2014) and were mostly inter-regional (Duncan et al., 2014). Therefore, the direction of correlation may be specific to the network under investigation and its physiological status under rest and stimulation. Present results also showed that BOLD changes were strongly dependent on resting state metabolic conditions in S1BF.

### Thalamocortical relationships between BOLD responses [Glu] and [GABA]

During TGN stimulation, GABA and Glu levels were uncorrelated within each structure and between structures. On the other hand, resting [Glu] and [GABA] in S1BF were correlated suggesting a strong interplay between these neurotransmitters in S1BF to maintain the EIB at stable levels as would be expected without specific brain activity. Such a relationship was not found in the thalamus and attributed to a lack of specificity since the region of interest taken into account encompassed several thalamic nuclei in order to increase SNR levels for single voxel MR ^1^H spectroscopy. Notably, the VOI emcompassed the reticular thalamic nucleus described as the only source of thalamic GABAergic inhibition. In addition, it receives inputs from the cerebral cortex but does not project to the cerebral cortex. The inclusion of this nucleus may explain differences between thalamic and cortical neurochemical profiles but also uncorrelated thalamic BOLD responses and resting thalamic GABA levels. Many studies suggested that RTN plays a significant role in thalamic gating, facilitating relevant stimuli and inhibiting others by increased inhibition of the information transfer via other relay nuclei. The shape and size of RTN make this important nucleus very difficult to avoid. Other techniques such as proton MR spectroscopic imaging (MRSI) (Seuwen et al., 2015) may be more adequate for future investigations while fMRS techniques coupled to optogenetic stimulation may help unraveling the exact contribution of this structure (Jurgens et al., 2012).

**Figure 6:**
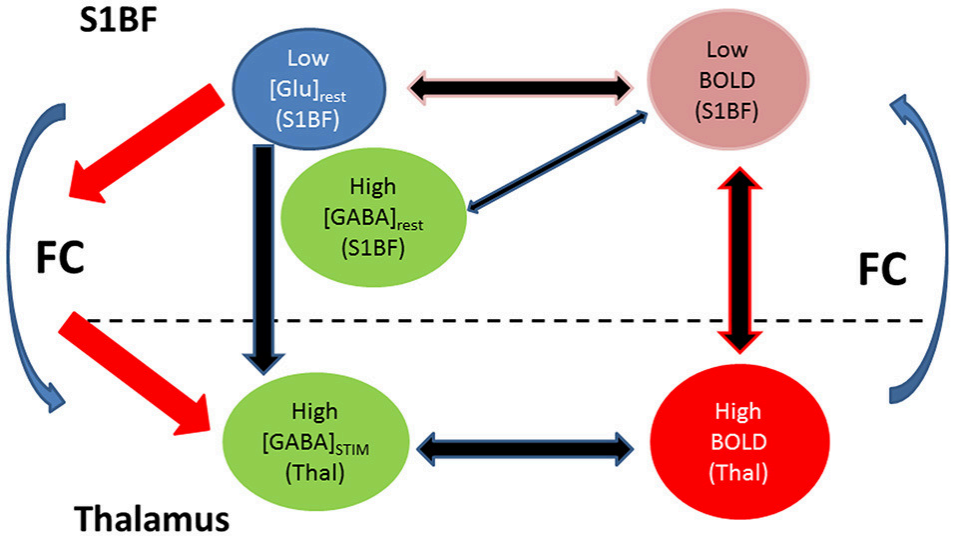
**Preliminary schematical overview of the Glutamate and GABA modulatory effects and functional connectivity on cortico-thalamo cortical loop in a healthy rat with low BOLD in S1BF (1–2%) under prolonged trigeminal nerve electrical stimulation**. Multi-regional investigations during brain activity may help understanding how neurotransmitters relate to functional neuroimaging signals and functional connectivity. Within each structure BOLD responses were modulated by within structure GABA and/or Glutamate levels. In the thalamus, BOLD responses were modulated by GABA during stimulation. These thalamic GABA levels demonstrated strong dependence on resting Glu levels in S1BF. Glutamatergic and GABAergic processes modulate thalamocortical and corticothalamic functional connectivity (FC). We postulate that these processes may involve regulation of neuronal synchrony between thalamus and cortex.

A significant negative correlation between BOLD responses measured in both S1BF and thalamus was found. In the literature, weak or absent thalamic BOLD responses with regards to forepaw or barrel cortex activations were reported in the past (Esaki et al., 2002; Keilholz et al., 2004; Zhao et al., 2008; Mishra et al., 2011; Devonshire et al., 2012). Thalamic BOLD responses are usually expected to be weaker than cortical ones due to generally higher GABA levels linked to higher densities of GABAergic neurons (Mishra et al., 2011; Devonshire et al., 2012; Tiwari et al., 2013). Here, accordingly, thalamic GABA levels were higher than in S1BF at rest and during stimulation but there was no significant increase of mean GABA levels in the thalamus upon TGN stimulation. Moreover, thalamic BOLD responses and GABA levels were uncorrelated. While these findings may be attributed to the inclusion of RTN, rats with higher resting S1BF GABA levels had high amplitude BOLD responses in the thalamus correlated to low amplitude BOLD responses in S1BF. Moreover, [Glu]_rest_ in S1BF were negatively correlated to [GABA]_stim_ in the thalamus. Therefore, rats with low Glu levels and high resting GABA levels in S1BF demonstrated lower BOLD responses in S1BF. Consequently, the release of high GABA concentrations in the thalamus could be interpreted as a way to overcome the higher excitatory activity supported by significantly increased thalamic Gln levels during TGN stimulation and thus balancing the decreased Glu levels and inducing strong BOLD thalamic activation. In the thalamus, [Gln] increased significantly during TGN stimulation while [Glu] decreased without significance. Interestingly, identical changes were reported by Xu et al. (2005) in the forepaw cortex of rats and were attributed to an augmented release of Glu and a rapid conversion into Gln through the Glu-Gln cycle between glutamatergic neurons and astrocytes. (Hirata and Castro-Alamancos, 2010) suggested that neuromodulators can control the synaptic activity of thalamocortical and corticothalamic cells and therefore control the state of their respective targets without being released there directly, thereby ensuring that both structures are under specific conditions essential for a proper connectivity. This is furthermore supported by studies suggesting the control of the synchrony of neuronal activity by regional EIB (Kapogiannis et al., 2013) and its correlation with functional connectivity. Therefore, the present study confirmed the interest in investigating relationships between neurotransmitters within the thalamus and the barrel cortex and between these two regions as potential predictors of functional connectivity, which could be of interest for studying pathologies such as absence epilepsy or schizophrenia. Duncan et al. (2014) in their review pointed at the fact that most studies looking at the roles of GABA and glutamate in brain function remain at the correlational stage while most of them include small numbers of subjects thus limiting the validation of findings. In the present work, functional connectivity was conducted on 8 rats only and showed high variability across this population (Figures S1, S2). At this stage, findings remain only indicative and should be further validated with more robust resting-state connectivity analysis. Figure 6 illustrates a preliminary schematical overview of the Glutamate and GABA modulatory effects and functional connectivity on cortico-thalamo cortical loop in a healthy rat.

## Limitations and perspectives

### Statistical analysis and multiple comparisons

In the text, correlations between either BOLD responses and metabolites or between metabolites were reported as being significant for uncorrected *p*-values. Upon application of corrected *p*-values as stated in Materials and Methods most correlations did not pass significance level which was attributed to the small number of animals included in the study. As a consequence of this, the correlations reported here point at interesting effects that remain to be validated. Nevertheless, to date, most of the correlation studies involving BOLD-metabolite relationships were not corrected for multiple comparisons. Table 3 summarizes various results from these studies with Pearson's correlation coefficients, p values and subject numbers. The column “statistics” mentions the statical test applied to the correlation. Unfortunately, these studies were mainly performed in humans. The number of subjects taken into account was in general similar to the number of rats used in the present study while significant negative correlation of brain activity measures with resting GABA were consistently found across studies including this one but not correlations with Glu levels. Although significance levels were not always adjusted, all these studies demonstrate that BOLD variability can be explained by GABA levels at least in cortical areas and the negative relationship between BOLD-resting GABA is a hallmark of neuronal activity. Here, the robustness of the correlation study was further confirmed by the Spearman-Rho correlation analysis and the multiple linear regression analysis. Although we delibaretely added Glutamate and GABA levels estimates with lower reliability (CRLB <=40%) from seven animals, we found that F statistics were significant while calculation of distance values (Cook's and leverage) increased the robustness of the analysis compared to a lower sample size of 15 animals. In addition, these analyses emphasized the roles of S1BF resting glutamate and GABA levels. The multiple linear regression analysis further demonstrated the need for higher sample sizes to validate metabolic correlations in rats but also pointed at the need for improved methods for associating MRS values as the interpretation of multiple linear regression models with an increased number of variables becomes extremely complicated.

**Table 3:** **BOLD-metabolites relationships in various human studies**.

**Study**	**Brain area**	**Correlation**	**Pearson's R**	***P*-value**	**Number of participants**	**Statistics**
Bednařík et al., 2015	Visual cortex	BOLD vs. water linewidth change	0.93	0.0001	11(15)	Not explained
		BOLD vs. ΔGlu	0.93	0.001	11(15)	Not explained
		BOLD vs. ΔLac	0.65	0.003	11(15)	Not explained
		BOLD vs. GABA	−0.65	0.043	11(15)	Not explained
Donahue et al., 2010	Visual cortex	BOLD vs. GABA	−0.7	*P* < 0.05	12	two tailed *P*-value
		VASO vs.GABA	−0.71	*P* < 0.05	12	
		ASL vs.GABA	0.41	*P* > 0.05	12	
		CBF vs.GABA	0.65	*P* < 0.05	12	
Muthukumaraswamy et al., 2012	Visual cortex	BOLD vs. GABA	−0.64	*P* < 0.05	10(12)	Not explained
		BOLD vs.γ frequency	−0.87	*P* < 0.01	10(12)	Not explained
Duncan et al., 2011	Supragenual and anterior	BOLD sgACC vs. (Glu+Gln in pgACC)	Not reported	Not reported	12	Spearman's rho, two tailed
	Cingulate cortex					
Enzi et al., 2012	Anterior cingulate cortex	BOLD vs. (Glu+Gln)	range: −0.436 to 0.853	<0.001–0.432	15–17	Paired *t*-tests
	Left anterior insula	BOLD vs. (Glu+Gln)	range: −0.357 to 0.09	>0.1	16–18	
Kühn et al., 2016	Inferior frontal gyrus	BOLD vs. GABA	−0.169; −0.352	0.152; 0.502	18	Not explained
		BOLD vs. Glu	0.144; 0.172	0.569; 0.494	18	Not explained
	Anterior cingulate cortex	BOLD vs. GABA	−0.522; −0.475	0.026; 0.046	18	Not explained
		BOLD vs. Glu	−0.27; −0.241	0.279; 0.346	18	Not explained
Stagg et al., 2011	Motor cortex	Cortical excitability vs. Glu	0.803	0.015	12	Bonferroni correction
		Cortical excitability vs. GABA	−0.79	0.018	12	Bonferroni correction
		Glx/cr vs. FC in PgACC and AI	−0.51 (Dep)	0.031		
Horn et al., 2010	pgACC, AI		0.15	0.49	18–22	Not explained

### Study design

One of the main pitfalls of the present study was its sequential design that unfortunately did not allow simultaneous assessment of BOLD, thalamic and S1BF metabolites. Using magnetic MRSI techniques such as FIDLOVs (Seuwen et al., 2015) which would at the same time contribute to preserve SNR levels and time may help while there is definitely a need for increased targeting at smaller structures with higher SNR levels for an increased specificity of studies as mentioned earlier for RTN or in pathologies (Pan et al., 2015). Finally, BOLD responses may be measured directly from fMRS acquisitions using changes in linewidths of NAA or total Creatine peaks as a result of activation which would also decrease scanning time. Again these measurements require high SNR levels per subject and require validation against standard BOLD acquisitions. In order to increase SNR levels for the temporal assessment of metabolite changes during TGN stimulation (not presented here), more fids were acquired increasing significantly the scanning time. Adaptation/habituation effects may therefore have been induced in the present study and will certainly need to be taken into account in future investigations.

### Impact of anesthesia

In the present study, fMRS findings in the cortex of rats under α-chloralose anesthesia were similar to findings obtained in humans (Schaller et al., 2014; Bednařík et al., 2015). BOLD-GABA and BOLD-ΔLac correlations had similar directions. Nevertheless, the impact of anesthesia on the modulatory effects of Glu and GABA on thalamic and S1BF BOLD responses as well as resting-state functional connectivity cannot be neglected (Williams et al., 2010).

## Conclusion

In rats under α-chloralose anesthesia, barrel cortex fMRS findings and association of these findings with BOLD responses were in accordance with recent results obtained in the human visual cortex. These results suggested consistent regulatory roles of both glutamate and GABA on cortical neural activity across species. Within the thalamus, neurochemical profiles during TGN stimulation differed from those obtained in the barrel cortex. The correlation study conducted within and between cortical and subcortical structures suggested a complex interplay between glutamatergic and GABAergic modulations of the cortico-thalamo-cortical loop. In addition, preliminary results suggested regulation of functional connectivity between thalamus and cortex by excitatory-inhibitory neurotransmitters. These results will need to be followed up with larger population sizes to demonstrate their validity.

To the best of our knowledge, this is the first study investigating two interconnected brain regions during both rest and activation periods using fMRS in rodents. Although in its early stage, the proposed methodology allowed investigating the different contributions of excitatory and inhibitory neurtransmitters on neuronal activity indirectly measured with BOLD in the thalamus and barrel cortex, which could benefit investigations of diseases affecting thalamo-cortical neurotransmission (Autism, Parkinson's disease, Gilles de la Tourette syndrome, cardiac dysfunction…).

## Author contributions

NJ conceived and designed the project. NJ performed experiments and analyzed the data. NJ wrote the manuscript. SS contributed to the discussion and revised the manuscript.

## Funding

This study was supported by the Centre d'Imagerie BioMédicale (CIBM) of Ecole Polytechnique Fédérale de Lausanne (EPFL), the University of Lausanne (UNIL) and the Foundations Leenards et Jeantet. SS was funded by a National Competence Center Biomedical Imaging grant (NCCBI).

### Conflict of interest statement

The authors declare that the research was conducted in the absence of any commercial or financial relationships that could be construed as a potential conflict of interest.

## Supplementary material

Figure S1 **Resting-state BOLD fMRI. (A,B)** BOLD T map overlaid over gradient echo EPI and showing bilateral resting-state BOLD both in the barrel cortex **(A)** and the thalamus **(B)** during long stimulation periods (2 min OFF–10 minON–2 minOFF) and short stimulation periods (30 s OFF–30 s ON…). **(C)**. Correlations between left and right hemisphere time courses for bilateral seeds were increased after long stimulations in individual animals but did not reach significance at the population level.

Figure S2 **Influence of [Glu] and [GABA] during rest and stimulation on cortico-cortical and thalamo-thalamic functional connectivities. (A,B)** Highest positive Pearson's correlation coefficients in both contralateral and ipsilateral S1BF (*r* = 0.57–0.67), **(A)** were found between stimulated Glu levels and *T*-values while cluster numbers were mainly correlated to GABA levels at rest **(B)**. **(C)** Thalamo-thalamic FC was negatively correlated to stimulated GABA levels **(D–G)** At rest, thalamic GABA levels were positively correlated to cluster numbers (*r* = +0.57. **(D)** A positive relationship between stimulated thalamic Glu levels and S1BF *T*-values (*r* = 0.69). **(E)** was observed but a negative one was observed between stimulated thalamic GABA levels and both *T*-values and Cluster numbers (*r* = −0.57, −0.56 respectively) **(F,G)**. **(H)** Negative correlation between cortico-thalamic FC and glutamate at rest.

## Abbreviations

fMRS: Functional Magnetic Resonance Spectroscopy
BOLD: Blood Oxygen Level Dependent
TGN: trigeminal Nerve
S1BF: Primary somatosensory Barrel Field cortex
[Glu]: glutamate concentration
[GABA]: γ-amminobutyric acid concentration
[Lac]: Lactate
[Gln]: Glutamine
VPM: ventral posteromedial thalamic nucleus
POm: Posteromedian Thalamic Nucleus
VOI: Volume of Interest
VASO: vascular space occupancy
CBF: cerebral blood flow
fWE: family wise error.
