# Supplementary figures and images for: Investigating the Role of Glutamate and GABA in the Modulation of Transthalamic Activity: A Combined fMRI-fMRS Study
Source: Front Physiol. 2017 Jan 31;8:30. doi: 10.3389/fphys.2017.00030 (PMC5281558; doi:10.3389/fphys.2017.00030)

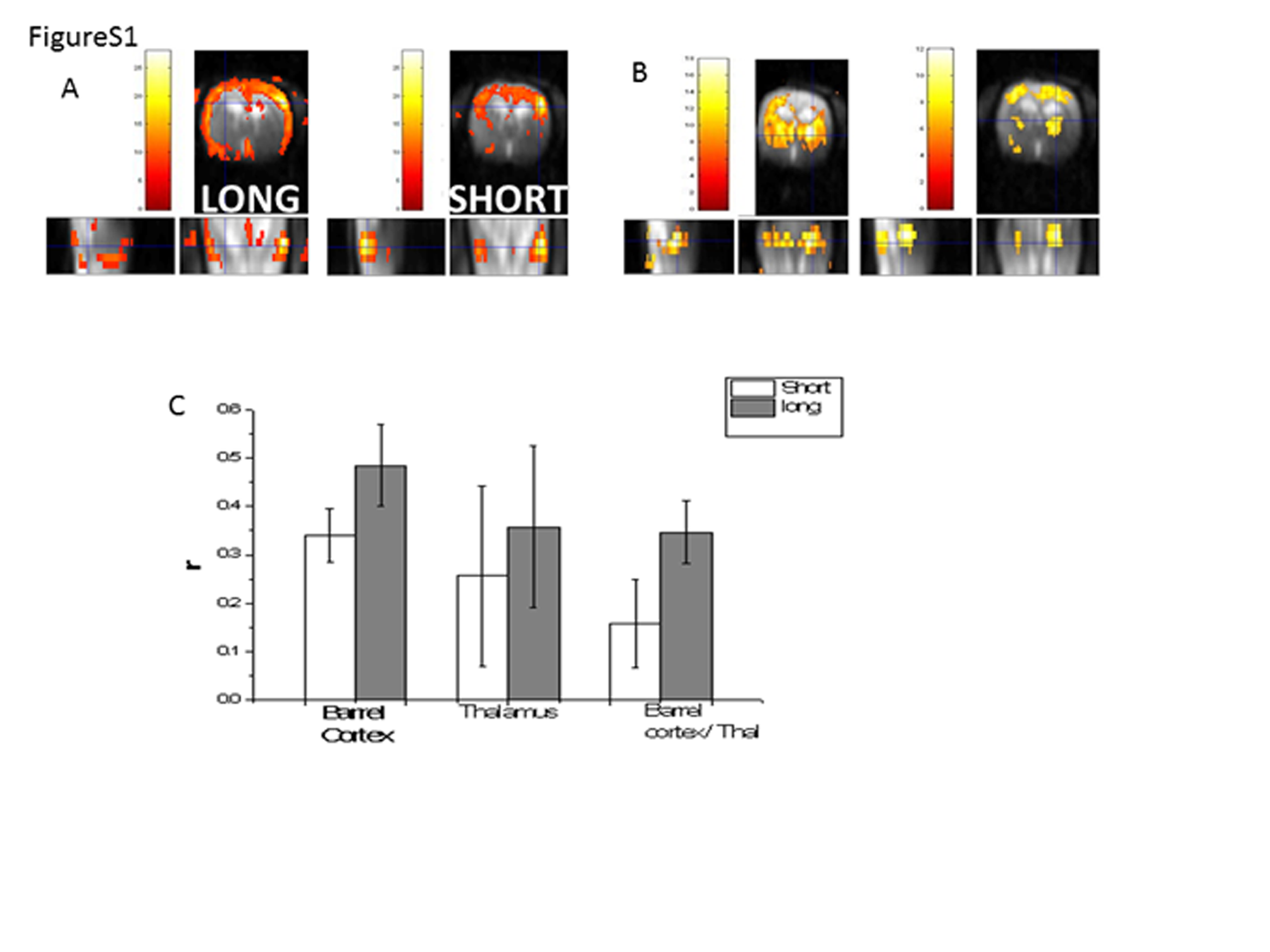

Supplement: Figure S1 — Resting-state BOLD fMRI. (A,B) BOLD T map overlaid over gradient echo EPI and showing bilateral resting-state BOLD both in the barrel cortex (A) and the thalamus (B) during long stimulation periods (2 min OFF–10 minON–2 minOFF) and short stimulation periods (30 s OFF–30 s ON…). (C). Correlations between left and right hemisphere time courses for bilateral seeds were increased after long stimulations in individual animals but did not reach significance at the population level. [file Image1.TIF]

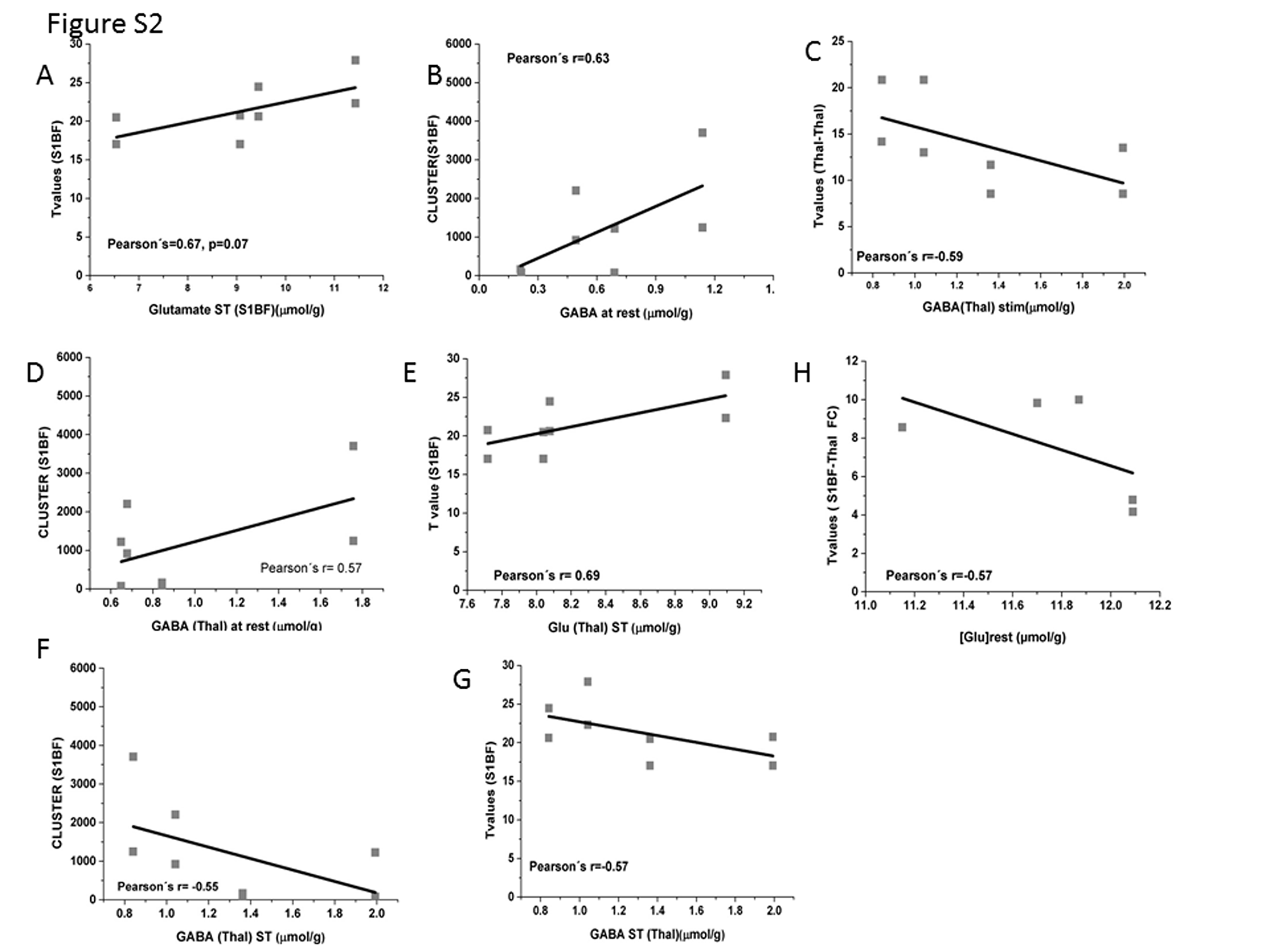

Supplement: Figure S2 — Influence of [Glu] and [GABA] during rest and stimulation on cortico-cortical and thalamo-thalamic functional connectivities. (A,B) Highest positive Pearson's correlation coefficients in both contralateral and ipsilateral S1BF (r = 0.57–0.67), (A) were found between stimulated Glu levels and T-values while cluster numbers were mainly correlated to GABA levels at rest (B). (C) Thalamo-thalamic FC was negatively correlated to stimulated GABA levels (D–G) At rest, thalamic GABA levels were positively correlated to cluster numbers (r = +0.57. (D) A positive relationship between stimulated thalamic Glu levels and S1BF T-values (r = 0.69). (E) was observed but a negative one was observed between stimulated thalamic GABA levels and both T-values and Cluster numbers (r = −0.57, −0.56 respectively) (F,G). (H) Negative correlation between cortico-thalamic FC and glutamate at rest. [file Image2.TIF]
