# Supplementary material for: Investigating the Role of Glutamate and GABA in the Modulation of Transthalamic Activity: A Combined fMRI-fMRS Study
Source: Front Physiol. 2017 Jan 31;8:30. doi: 10.3389/fphys.2017.00030 (PMC5281558; doi:10.3389/fphys.2017.00030)
Supplement: Supplementary file 3 [file DataSheet1.docx]

**Supplementary data**

**Materials and Methods**

**Resting-state BOLD fMRI**

8 rats out of the 15 used for fMRS S1BF and thalamus were used for resting-state BOLD fMRI. Only 8 animals could be analyzed –data in 4 animals were discarded due to problems with the scanner for 2 animals and degraded physiological conditions for 2 other animals (Blood pH <7.0).

Following FASTMAP shimming, single–shot gradient-echo EPI images were acquired during short (30sOFF-30sON-…; 1Hz; 2mA) and prolonged (2minOFF-10minON-2minOFF, 1Hz, 2mA) electrical right trigeminal nerve stimulations of S1BF in order to verify both responsivity of our rats as well as whether functional connectivity (FC) between the barrel cortex (S1BF) and the thalamus (Thal) should be different after short or prolonged S1BF stimulations. Resting-state BOLD fMRI was conducted after each type of stimulation. The data analysis was conducted as in De Groof et al (2013). After realignment, slice timing and smoothing of functional images with SPM8, seed regions were drawn bilaterally on S1BF and Thal using the REST toolbox (the Resting-State fMRI Data Analysis Toolkit (REST 1.7; <http://www.restfmri.net/>)) to extract mean BOLD time courses that were de-trended and low pass-filtered (0.01-0.1Hz). FC maps were generated in SPM8 by comparing each seed time course with time courses of all other voxels within the rat brain using linear regression with the time course of the seed as covariate. T value maps were generated using a family wise error pvalue <1e-06 as a threshold. Comparisons between cluster numbers and T values in bilateral S1BF regions were performed for short and long stimulation conditions (Paired t-test, p<0.05). In addition, [Glu] and [GABA] estimated in S1BF and Thalamus during rest and stimulation periods from proton spectra previously acquired were correlated to the Tvalues and cluster numbers. Correlations between seed time courses and between metabolites and FC parameters were assessed using the Pearson´s correlation coefficient as described in Materials and Methods.

**Results**

**Resting-state functional connectivity**

FC maps indicated increased cluster numbers and hence increased FC after long stimulation periods mainly across the cortex (**Fig.S1A**) but also in thalamic regions (**Fig.S1B**). No significant differences were found between mean T values for short and long stimulations and between cluster numbers although the latter resulted from large differences across animals. Correlations between left and right hemisphere time courses for bilateral seeds were also increased after long stimulations in individual animals but did not reach significance at the population level (**Fig. S1C**).

Finally, the influence of GABA and Glu on cortico-cortical FC was evaluated by correlating these metabolites during both rest and activation conditions to T values and cluster numbers estimated from FC maps. In the present study we assumed that the T values and cluster numbers obtained following bilateral cortical and thalamic cross-correlations represented functional connectivity parameters (FC). Highest positive Pearson´s correlation coefficients in both contralateral and ipsilateral S1BF (r=0.57-0.67) (**Fig. S2A**) were found between stimulated Glu levels and Tvalues while cluster numbers were mainly correlated to GABA levels at rest (**Fig.S2B**). Thalamo-thalamic FC was negatively correlated to stimulated GABA levels (**Fig.S2C**). Using the same methodology, the influence of thalamic GABA and Glu levels at rest and during stimulation was also investigated. At rest, thalamic GABA levels were positively correlated to cluster numbers (r=+0.57, **Fig.S2D**). A positive relationship between stimulated thalamic Glu levels and S1BF Tvalues (r=0.69) (**Fig.S2E**) was observed but a negative one was observed between stimulated thalamic GABA levels and both Tvalues and Cluster numbers (r=-0.57, -0.56 respectively) (**Fig.S2F-G**).

**Discussion**

**Mediation of thalamocortical functional connectivity by glutamate and GABA**

Assuming that cluster numbers represent a measure of the spatial extent of FC and Tvalues represent the strength of FC, correlations with cortical Glu and GABA measured at rest and during stimulation periods showed that resting GABA levels mediated the spatial distribution of cortico- cortical FC whereas the strength of cortico- cortical FC was mediated by [Glu]_stim_. Similarly, thalamic GABA levels at rest mediated the spatial extent of cortico-cortical FC while balanced effects of thalamic [Glu]_stim_ and [GABA]_stim_ were related to both strength and spatial extent of cortico-cortical FC. Interestingly, high GABA levels measured during stimulation periods in the thalamus correlated with decreased levels of FC within both S1BF and thalamus as well as between S1BF and thalamus. Moreover, a preliminary negative correlation between S1BF [Glu]_rest_ and corticothalamic FC appeared to confirm an increased corticothalamic FC in rats with the lowest resting S1BF Glu levels (**Fig. S2H**). These correlated with lowest S1BF BOLD responses but highest thalamic BOLD responses. These findings reinforce the hypothesis of inter-regional neuronal synchrony being modulated by EIB (Kapogiannis et al., 2013, Duncan et al., 2014) but will need to be validated in more animals.
